# Maternal Weight Gain during Pregnancy and the Developing Autonomic Nervous System—Possible Impact of GDM

**DOI:** 10.3390/nu14245220

**Published:** 2022-12-07

**Authors:** Louise Fritsche, Julia Hartkopf, Julia Hummel, Dorina S. Löffler, Hajime Yamazaki, Hans-Ulrich Häring, Andreas Peter, Andreas L. Birkenfeld, Robert Wagner, Andreas Fritsche, Hubert Preissl, Martin Heni

**Affiliations:** 1 Helmholtz Center Munich, Institute for Diabetes Research and Metabolic Diseases at the University of Tübingen, 72076 Tübingen, Germany; 2 German Center for Diabetes Research (DZD), 85764 Neuherberg, Germany; 3 Division of Endocrinology and Diabetology, Department of Internal Medicine 1, University Hospital Ulm, 89081 Ulm, Germany; 4 Department of Internal Medicine, Division of Endocrinology, Diabetology, Nephrology, Eberhard Karls University Tübingen, 72076 Tübingen, Germany; 5 Section of Clinical Epidemiology, Department of Community Medicine, Graduate School of Medicine, Kyoto University, Kyoto 606-8507, Japan; 6 Institute for Clinical Chemistry and Pathobiochemistry, Department for Diagnostic Laboratory Medicine, University Hospital Tübingen, 72076 Tübingen, Germany; 7 Institute for Clinical Diabetology, German Diabetes Center (DDZ), Leibniz Center for Diabetes Research at Heinrich-Heine University, 40225 Düsseldorf, Germany

**Keywords:** gestational diabetes mellitus, gestational weight gain, autonomic nervous system, heart rate variability

## Abstract

Objective: The intrauterine environment is known to affect the offspring’s long-term risk for obesity and diabetes. Previous data show that maternal metabolism and gestational weight gain (GWG) are associated with fetal autonomic nervous system (ANS) function, which can be assessed with heart rate variability (HRV). We investigated whether this association is also present in 2-year-old children and addressed the impact of gestational diabetes (GDM). Research design and methods: We examined the 2-year-old offspring of mothers who had undergone a 5-point, 75 g oral glucose tolerance test during pregnancy. To assess HRV, a 10-minute ECG was recorded, and time domain and frequency domain parameters were analyzed. Body composition was assessed using bioelectrical impedance testing. Results: We examined 67 children (33 girls, 34 boys), 30 of whom were born to mothers with treated GDM and normoglycemic pregnancies (NGT), respectively. No differences were found between the groups with regard to birth weight, weight at the age of 2 years, and body fat content. We observed that GWG was associated with heart rate and HRV, indicating that children of mothers with low GWG had a lower parasympathetic tone. This association was detected in NGT-exposed—but not in GDM-exposed—children. HR and HRV correlated with body fat and fat-free mass in children from normoglycemic pregnancies only. Conclusion: We found that the impact of maternal GWG on offspring ANS function was missing in the presence of treated GDM. The balance of the ANS was related to offspring body composition in children from NGT pregnancies only. Our results suggest that maternal weight gain during pregnancy has a critical impact on the developing ANS, which might be disturbed in the presence of GDM.

## 1. Introduction

The autonomic nervous system (ANS) is a crucial regulator of major physiologic functions throughout the entire organism. It consists of the two counteracting branches known as the sympathetic and parasympathetic branches. These innervate the smooth musculature of all organs, the heart, and glands. The balance between the two ANS branches is altered by a number of pathological conditions, including obesity and metabolic diseases, such as type 2 diabetes [1].

ANS formation and development are believed to take place in a sequential manner [2,3], with an increase in parasympathetic activity from the end of the second trimester [4]. On the basis of the framework of Developmental Origin of Health and Disease (DOHaD [5]), it is assumed that not only genetics but also the intrauterine environment impacts fetal development, including the normal development of the ANS. This is thought to have consequences for the future health of the offspring via fetal programming, epigenetic modification, and imprinting.

The availability of maternal substrates designated for the fetus is crucial during pregnancy and has an impact on the offspring’s later life well into adulthood. Despite impaired fetal growth and low birth weight in the event of poor maternal nutrition, undernutrition, and insufficient gestational weight gain (GWG), such children are at risk to subsequently develop obesity, type 2 diabetes mellitus (T2DM), and cardiovascular disease (CVD) [6]. Conversely, children who are exposed to an overabundance of maternal substrates in utero (glucose, lipids and amino acids) often experience fetal overgrowth. Such an environment is present, for example, in the cases of gestational diabetes (GDM), high pre-gestational BMI, or excessive GWG. Children of mothers with disproportionally high GWG or GDM are also prone to childhood overweight and impaired glucose metabolism, both of which increase the risk of obesity, T2DM, and CVD in adulthood [7]. GDM is defined as glucose intolerance first diagnosed during pregnancy. It develops when insulin resistance, which is physiologically increased during the second half of pregnancy [8] and which is necessary to facilitate sufficient nutrient flux to the fetus, cannot be met by adequate insulin secretion.

Furthermore, the intrauterine environment has a crucial impact on the ANS. The balance of sympathetic and parasympathetic action can be determined by measuring heart rate variability (HRV) and heart rate (HR). Lower HRV and higher HR are associated with overweight and metabolic diseases such as diabetes in children and adults [1,9,10]. HRV and HR can both be recorded non-invasively by electrocardiography (ECG). During the second half of a pregnancy and in the early neonatal phase, the ANS undergoes a critical phase of maturation [4], during which it might be susceptible to harmful intrauterine conditions caused by inadequate GWG due to under- and overnutrition. Indeed, we and others have previously shown that maternal factors such as pre-pregnancy weight, gestational weight gain [11,12], and gestational diabetes [13] can adversely affect HRV and HR in a developing child, even in utero. Inappropriate GWG therefore appears to have an impact on the developing ANS. Whether this persists after birth and how a differently programmed ANS could change postnatal weight trajectory and future risk of obesity or T2D remains unclear. Findings demonstrated that low birth weight is associated with a lower parasympathetic tone in 5–14-year-old children, albeit GWG was not addressed in this study [14].

We therefore investigated whether GWG was still linked to ANS balance in the offspring two years after birth. On the basis of our previous findings in utero [11,13], we hypothesized that the presence of GDM during pregnancy might have an additional impact. Furthermore, we investigated the relationship of ANS to offspring body weight.

## 2. Materials and Methods

### 2.1. Participants

Offspring from mothers who had participated in the ongoing prospective multicenter PREG study in Tübingen (clinical trials identifier NCT04270578) were examined at 2 years of age. Their mothers had been enrolled into the PREG study during pregnancy between gestational weeks 24 + 0 and 31 + 6. The detailed methodology was described recently [15]. In short, PREG study participants with singleton pregnancies underwent a diagnostic 5-point oral glucose tolerance test (OGTT) with 75 g of glucose following an overnight fast of 12 h. GDM was diagnosed in accordance with the recommendations of the International Association of the Diabetes and Pregnancy Study Groups (IADPSG) published in 2010 [16]. GDM was treated strictly in accordance with national guidelines [17], with frequent nutritional counseling including food diaries, self-measurement of blood glucose in the fasting and postprandial state, lifestyle modification, and basal/bolus insulin therapy, when indicated. A subset of 11 mothers with a confirmed GDM diagnosis documented in medical records was enrolled after pregnancy. Pregnancy and birth outcomes were collected from medical records and questionnaires. All study participants provided written and informed consent. The study protocol was approved by the Ethics Committee of the University Hospital Tübingen and was conducted in accordance with the Declaration of Helsinki.

### 2.2. Measurements and Calculations

#### 2.2.1. Children

The children were examined in the late morning in the presence of a parent. Anthropometric data were taken, and body composition was assessed with bioelectrical impedance testing using an Akern BIA101 (SMT medical GmbH & Co, Würzburg, Germany). Body fat and fat-free mass were calculated using the method described by Goran et al. [18]. BMI-for-age z-scores were calculated in accordance with the recommendations of the World Health Organization (WHO) [19] using the igrowup-package for R.

A ten-minute ECG was recorded at rest in the supine position with BIOPAC MP30 (BIOPAC Systems Inc, Goleta, CA, USA) at a sampling rate of 1000 Hz. To ensure a resting state, the parent was placed next to, and instructed to read a book to, the child. Heart rate and heart rate variability parameters were calculated with the KUBIOS HRV software V.2.2. We analyzed standard measures for HRV [20] from the time domain (beats per minute (BPM), root mean square of successive differences (RMSSD)), standard deviation of RR-Intervals (SDNN), and the frequency domain (low frequency (LF), high frequency (HF), and LF/HF). An overview of the assessed HRV parameters and their associated autonomous function is provided in Supplementary Table S1.

#### 2.2.2. Mothers

Office-recorded maternal weight data were taken from the Germany-specific maternity log, which was issued and kept for every pregnant woman by her gynecologist. Total gestational weight gain was estimated by calculating the difference between the first weight recorded in the first trimester and the last weight recorded prior to delivery. Weight gain from the last trimester up until delivery was calculated from the weight documented for gestational weeks 24 to 28. Classification of GWG as insufficient, appropriate, or excessive was carried out depending on the pre-gestational BMI in accordance with the Institute of Medicine (IOM) recommendations from 2009 [21] (Supplementary Table S2). Insulin sensitivity was calculated with the NEFA-insulin sensitivity index [22]. Maternal physical activity at the time of OGTT was assessed with the habitual physical activity index [23]. Breastfeeding practices were assessed retrospectively with a questionnaire [15] that recorded any breastfeeding (exclusive and partial). 

### 2.3. Statistical Analysis

Statistical analysis was carried out with R Version 4.1.0. Data are presented as mean (SD), median [IQR], or as counts (%). All variables were tested for normal distribution using the Shapiro–Wilk test and log-transformed if necessary. Group differences (GDM vs. control) were tested with t-test, Kruskal–Wallis-test, and Chi^2^-test. We analyzed the effect of GWG on HR and HRV with multivariable linear regression adjusted for pre-gestational BMI and offspring sex separately for GDM and control. We tested the interaction of GWG and GDM status by including the interaction term (GWG X GDM status) in the same model, with the main effects included in the model as well. A *p*-value < 0.05 was considered statistically significant, and *p* < 0.1 was considered a trend.

## 3. Results

### 3.1. Maternal and Offspring Characteristics

In this study, 67 children were examined (34 male, 33 female). GDM was diagnosed in 30 of their mothers, eight (26.7%) of whom received insulin therapy. A total of 37 women had normoglycemic pregnancies (NGT). Maternal age did not differ between the two groups. Maternal characteristics for women with and without GDM are shown in Table 1. Pre-gestational BMI and gestational weight gain differed significantly: Women who developed GDM during pregnancy were heavier before conception but had lower total and third trimester GWGs. However, the proportion of insufficient/appropriate/excessive GWG did not differ between groups (Table 1).

The clinical characteristics of the offspring at birth were similar between NGT and GDM with regard to gestational age at birth, birth length, birth weight, and head circumference (Table 1). 

The children were re-examined at the mean age of 25.4 (±1.7) months. Neither absolute weight nor BMI-for-age z-score nor body fat content differed between groups (Table 1).

### 3.2. Maternal Gestational Weight Gain Is Associated with Offspring ANS Function 

We first analyzed the relationship between maternal gestational weight gain and offspring ANS function in the entire cohort of 2-year-old children, regardless of GDM status. Total gestational weight gain was associated with lower HR, increased RMSSD, increased HF power, increased SDNN, and decreased LF/HF ratio (all adjusted for pre-gestational BMI, offspring age, and sex; Supplementary Figure S1), indicating lower parasympathetic activity in children of mothers with low GWG and higher activity in those whose mothers had higher GWG.

**Figure 1:**
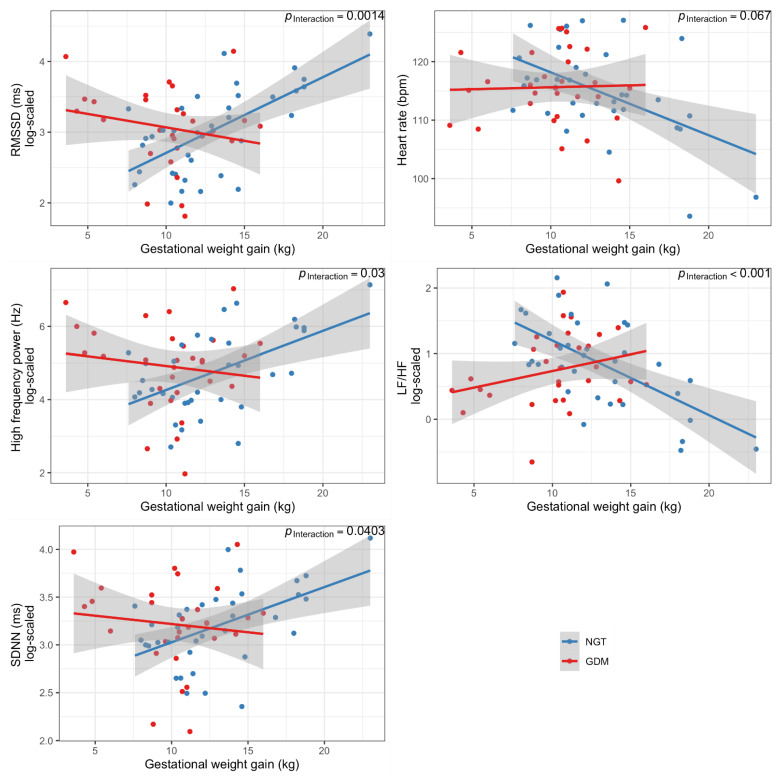
Association of total gestational weight gain with heart rate variability parameters of 2-year-old offspring in the whole cohort (by GDM status of the mother). RMSSD, root mean square of successive differences; SDNN, standard deviation of RR-Intervals; LF, low frequency; HF, high frequency; BPM, beats per minute.

GDM status per se was not associated with offspring ANS function (Supplementary Table S3). However, there was a significant interaction between GDM status and GWG on HRV (Table 2, Figure 1): children from mothers with a normoglycemic pregnancy showed a strong association of GWG with HRV and HR, whereas this was absent in the children who had been exposed to GDM (Figure 1, Table 2).

GDM was diagnosed in gestational week 27.2 (±1.9) and subsequently treated until delivery, which probably affected GWG in the third trimester of pregnancy. We therefore separately analyzed the effect of (i) GWG up until GDM diagnosis and (ii) third-trimester-GWG (i.e., GWG after diagnostic OGTT diagnosis) on HRV. While there was no association with GWG until the diagnostic OGTT, only the third-trimester GWG had an association with HRV parameters in NGT offspring, but not GDM exposed children (Supplementary Table S3). The type of GDM treatment (diet vs insulin therapy) and last-trimester GWG showed no association with offspring ANS function (adjusted for offspring age and sex). 

We next investigated potential influencing factors. Neither maternal physical activity nor parity nor triglyceride levels at the time of the OGTT were associated with HRV in the 2-year-old offspring (adjusted for gestational age at OGTT, offspring age, and sex). In addition, no association with maternal insulin sensitivity was determined at the time of OGTT (adjusted for gestational age, BMI at OGTT, offspring age, and sex). Pre-pregnancy BMI per se was not associated with offspring ANS function.

### 3.3. Offspring ANS Function Is Associated with Body Composition Only in Children from Mothers with Normoglycemic Pregnancy

**Figure 2:**
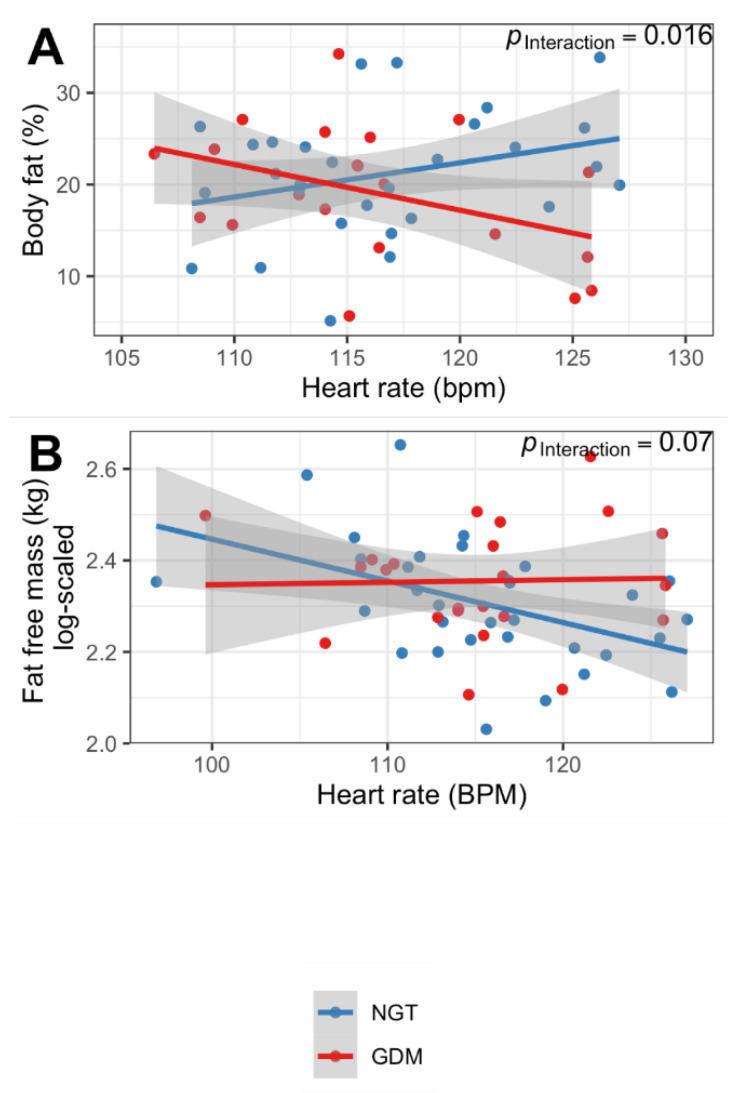
Association of heart rate with percent body fat (**A**) and fat-free mass (**B**) in 2-year-old children. Interaction of GDM status and heart rate.

We also examined how HR and HRV were associated with offspring weight and body composition. We detected a positive association between HR and body fat content in children born from pregnancies with NGT. By contrast, this association was not found in the children who had been exposed to GDM (*p*_Interaction_ = 0.016 (interaction of GDM status x HR), Figure 2A). Heart rate and fat-free mass tended to be negatively associated in children from NGT mothers but not in the GDM group (*p*_Interaction_ = 0.07, Figure 2B). With regards to the other HRV parameters assessed, there was no statistically significant association between offspring body weight and body composition.

## 4. Discussion

We detected a link between maternal GWG and ANS function in two-year-old children. This finding was, however, limited to children of mothers with normal glucose metabolism during pregnancy. While the GWGs of NGT mothers were positively associated with parasympathetic tone in two-year-old children, no such association was detected in children exposed to GDM during pregnancy. Only in children from normoglycemic pregnancies was ANS function linked to body composition, with less body fat in children with higher parasympathetic tones.

Infants born to mothers with GDM were neither heavier nor larger at birth than infants born to mothers with normal glucose metabolism. The normal birth outcome in women with GDM in our study is probably due to the timely initiation of a tight treatment regime following GDM diagnosis, as also demonstrated in other cohorts [24]. The adverse consequences of insufficiently controlled GDM on offspring BMI and body composition are well-documented [25,26]. Nevertheless, residual consequences of a seemingly successfully treated GDM on long-term offspring metabolic health and body weight are less clear. Some studies reported no effect of treating mild GDM on obesity rates in prepubertal children [27,28]. In our cohort of 2-year-old children, BMI and body composition were comparable between children who had or had not been exposed to GDM, presumably due to strict GDM management. Nonetheless long-term effects may present later in childhood, possibly at onset of puberty [29]. 

Importantly, the development of body weight and body composition is regulated not only by hormonal and nutritional factors but also by the ANS. A differently programmed ANS due to these factors could have an impact later in life. It could promote the propensity toward overweight and unfavorable body composition in the offspring. 

Of note, maternal GWG is associated with parasympathetic activity in 2-year-old offspring. Children of mothers with low GWG displayed higher HR and lower HRV, indicating a less beneficial ANS profile with higher sympathetic than parasympathetic tone. We are not aware of any other studies that address the long-term effects of maternal weight gain during pregnancy on offspring ANS function two years postpartum. Comparable associations have been reported only for fetal HR and fetal HRV in a cohort of normoglycemic women [11]. One study evaluated the quality of maternal diet during pregnancy and reported reduced HRV in the 6-month-old offspring of mothers who consumed a prenatal diet with a low Healthy Eating Index (HEI), thus probably impacting GWG. The weight gain was, however, not analyzed [30]. Our data suggest that sufficient GWG is necessary for normal ANS development and that this process is disturbed in the presence of GDM. One study examining fetal HRV during an oral glucose tolerance test at 30 weeks gestation is line with this interpretation. In these measurements, GDM-exposed fetuses showed lower HRV than the control [13], albeit the effect of GWG was not assessed as an independent variable in the study. 

The rate of weight gain differs physiologically during pregnancy, with most maternal weight accumulation occurring in the third trimester, which is also a crucial phase of ANS maturation [3]. In our data, only this weight gain in the third trimester was associated with ANS function, whereas the earlier weight gain was unrelated. On average, the women with GDM in our study had both lower total and third trimester weight gains. There are two possible reasons for this: First, the pre-pregnancy BMI was higher in this group (as is also the case in most other studies [31]), and women with higher BMIs are routinely advised to adopt healthy lifestyles to prevent excessive GWG [21,32]. Second, the patients diagnosed with GDM underwent diet counseling and weight management, with an aim to prevent macrosomia and related birth complications. This approach simultaneously limited GWG. We cannot rule out the possibility that GDM treatment, resulting in lower GWG in the last trimester compared to normal glucose-tolerant women, contributed to the lack of any association between GWG and ANS function in children from mothers with GDM. However, the fact that the ratio of insufficient vs normal vs excessive GWG did not differ between both groups argues against this.

It is unclear as to which aspect of GWG affects ANS development. Maternal obesity prior and during pregnancy was reported to be negatively associated with infant HRV and HR [33]. This is in contrast to our finding of a lack of association between pre-pregnancy BMI and offspring ANS function. Furthermore, insulin sensitivity and triglycerides at the time of OGTT did not show any link to ANS function. Since no data other than maternal weight were available for this period, we were unable to analyze factors contributing exclusively to GWG in the last trimester. Further mechanistic research is required to identify the factors affecting the development of offspring ANS in utero.

The association of ANS function, and children’s body weight and body composition, has already been demonstrated in overweight and obese prepubertal children [34]. RMSSD and SDNN were positively, and LF/HF ratio negatively, associated with higher body weight and body fat measures, indicating lower parasympathetic tones in these children [34]. HR was positively associated with body fat and weight [34]. Similar findings were reported for weight in a cohort of normal and overweight/obese British school children [10] and adolescents [35]. Although none of the children in our cohort were overweight at 2 years of age, the association of HR with fat-free mass and with fat mass was still detectable in children who had not been exposed to GDM. To our knowledge, no other studies investigating the link between ANS function and body composition are available for children as young as in our study. Our results demonstrate that the normal connection of ANS and body composition is already present at an early age, i.e., while the ANS is still maturating [36]. Of note, this connection was not found in the GDM-exposed children, despite them having comparable weights and body compositions. We speculate that GDM exposure adversely affects the normal programming of offspring ANS and leads to the observed differences of ANS body composition associations. With regard to GDM exposure, high glucose levels are associated with lower HRV and higher HR in adults [37] and in children [38], and high fetal glucose levels such as those found in GDM might affect the maturating ANS either directly or indirectly via high fetal insulin levels [39] and fetal insulin resistance. 

Although we detected no differences in body weight or body fat between GDM-exposed children and the control, the difference in the configuration of the ANS in GDM-exposed children might be a predictor for future weight and body composition development, as suggested by the findings from adult studies [40].

Our study has limitations. We had no cases of uncontrolled GDM in our study and all GDM cases were tightly treated, resulting in normal birth outcomes. Treatment effects, therefore, cannot be excluded as an explanatory factor for our findings. Furthermore, we examined children from mothers with a high level of education, which is not representative of the general population. This might also contribute to the normal weight and BMI at 2 years of age. Furthermore, the eating behaviors of the mothers were not systematically evaluated in all participating women.

## 5. Conclusions

In conclusion, we discovered a link between maternal GWG and configuration of ANS that resulted in detectable effects on body adiposity in 2-year-old children. This connection was impeded in the presence of well-treated GDM. Children with dominant sympathetic activity had higher body fat content than children presenting higher parasympathetic tone. Our results point toward a critical impact of maternal weight gain during pregnancy on the developing ANS, with long-lasting consequences for body composition. This underlines the importance of sufficient GWG for long-term health. The disturbance of this normal adaptation in GDM could be a non-genetic mediator of metabolic risk from mother to child.

## Figures and Tables

**Table 1:** Maternal and offspring characteristics.

Characteristics	Control (*n* = 37)	GDM (*n* = 30)	*p*	*p*_adjusted_
Maternal age	34.92 (3.75)	35.33 (5.21)	0.707	-
Parity (%) Nulliparous Multiparous	20 (54.1) 17 (45.9)	23 (76.7) 7 (23.3)	*0.096*	-
Mode of delivery (%) Spontaneous C-section	29 (78.4) 8 (21.6)	13 (43.3) 17 (56.7)	0.007	-
Pre-gestational BMI (kg/m^2^)	22.7 [20.7, 27.7]	25.1 [22.6, 31.0]	**0.041**	-
Total gestational weight gain (kg)	12.0 [10.5, 14.6]	10.6 [8.9, 12.2]	**0.002**	-
Third trimester gestational weight gain (kg)	4.9 [3.5, 6.7]	3.8 [2.1, 4.9]	**0.021**	-
GWG category (%) Insufficient Appropriate Excessive	11 (31.4) 13 (37.1) 11 (31.4)	13 (43.3) 10 (33.3) 7 (23.3)	0.586	-
Educational level (%) A-level Secondary school diploma None	29 (78.4) 8 (21.6) 0 (0)	15 (50.0) 13 (43.3) 2 (6.7)	**0.021**	-
GDM Treatment (%) None Nutritional therapy Insulin therapy	-	1 (3.3) 21 (70.0) 8 (26.7)	-	-
Gestational age at OGTT ^#^	27.4 (2.1)	27 (1.6)	0.508	-
Insulin sensitivity (NEFA-ISI) ^#^	3.49 [3.11, 4.57]	2.48 [2.11, 3.26]	**0.003**	-
Triglycerides (mg/dL) ^#^	178 (86)	245 (104)	**0.013**	-
Habitual physical activity index ^#^	8.16 (1.51)	7.38 (1.44)	*0.075*	*-*
Birth outcome				
Sex (%) Female Male	20 (54.1) 17 (45.9)	13 (43.3) 17 (56.7)	0.464	-
Gestational age at birth (weeks)	39.37 (1.55)	38.61 (1.99)	*0.086*	-
Birth weight (g)	3360 [3100, 3610]	3360 [2985, 3633]	0.940	0.683 ^†^
Birth length (cm)	51 [49, 53]	51 [49, 53]	0.804	0.524 ^†^
Macrosomia (%) yes no	4 (10.8) 33 (89.2)	1 (3.3) 29 (96.7)	0.49	-
Breastfeeding (%) yes no	32 (86.7) 5 (13.5)	26 (86.7) 4 (13.3)	1.000	-
Follow up				
Offspring age (months)	25.19 (1.73)	25.70 (1.64)	0.223	-
Weight (kg)	12.54 (1.36)	13.10 (1.80)	0.149	0.43 ^‡^
BMI (kg/m^2^)	16.41 (1.11)	16.11 (1.23)	0.310	0.14 ^‡^
Body fat (%)	21.11 (6.8)	19.13 (7.3)	0.334	0.46 ^‡^
BMI-for-age z-score	0.42 (0.81)	0.13 (0.97)	0.179	-

Data are presented as means (SD), medians [IQR], and numbers (%). Group differences were tested with t-test for normally distributed variables, Kruskal–Wallis-test for non-normally distributed variables, and Chi^2^-test for categorical variables. Bold values indicate statistical significance, and italic values indicate statistical trend. GDM, gestational diabetes mellitus. ^#^ N = 56; ^†^ Adjusted for gestational age at birth and sex; ^‡^ adjusted for age and sex.

**Table 2:** Association of total gestational weight gain with heart rate variability and heart rate in 2-year-old children of mothers with and without GDM.

	Heart Rate (BPM)		RMSSD (ms)		SDNN (ms)		Low Frequency (LF) Power (m^2^)		High Frequency (HF) Power (m^2^)		LF_n. u.		HF_n. u.		LF/HF	
	B (95% CI)	*p*	B (95% CI)	*p*	B (95% CI)	*p*	B (95% CI)	*p*	B (95% CI)	*p*	B (95% CI)	*p*	B (95% CI)	*p*	B (95% CI)	*p*
All children Interaction GWG x GDM status	0.99 (−0.07, 2.05)	*0.0668*	−0.14 (−0.22, −0.05)	**0.0014**	−0.07 (−0.13, 0.00)	**0.0403**	−0.03 (−0.16, 0.09)	0.6054	−0.19 (−0.35, −0.02)	**0.0307**	3.34 (1.62, 5.07)	**0.0003**	−3.30 (−5.00, −1.59)	**0.0003**	0.15 (0.07, 0.24)	**0.0006**
Children whose mothers had NGT	−1.02 (−1.67, −0.37)	**0.0031**	0.11 (0.06, 0.15)	**0.0001**	0.06 (0.02, 0.09)	**0.0030**	0.05 (−0.02, 0.11)	0.1795	0.15 (0.06, 0.25)	**0.0019**	−2.39 (−3.43, −1.35)	**0.0001**	2.34 (1.31, 3.37)	**0.0001**	−0.11 (−0.16, −0.06)	**0.0002**
Children whose mothers had GDM	0.02 (−0.90, 0.94)	0.9721	−0.03 (−0.10, 0.05)	0.4420	−0.01 (−0.08, 0.05)	0.6947	0.01 (−0.11, 0.13)	0.8344	−0.03 (−0.19, 0.13)	0.7187	0.88 (−0.65, 2.41)	0.2482	−0.88 (−2.39, 0.64)	0.2466	0.04 (−0.03, 0.11)	0.2551

Effect sizes (95% CI) and *p*-values of multivariate linear regression models for the association of total gestational weight gain (GWG) with offspring heart rate (HR) and heart rate variability (HRV). Row 3 shows the values for the interaction of total GWG and GDM status with HR and HRV parameters, adjusted for pre-gestational BMI and offspring sex. Rows 4 and 5 show the values from the multivariate linear regression of total GWG, with HR and HRV parameters adjusted for pre-gestational BMI and offspring sex in groups of NGT and GDM separately. Bold values indicate statistical significance; values in italics indicate a statistical trend.

## Data Availability

All requests for data and materials will be promptly reviewed by the Data Access Steering Committee to verify whether the request is subject to any intellectual property or confidentiality obligations. Individual-level data may be subject to confidentiality. Any data and materials that can be shared will be released via a Material Transfer Agreement.

## Supplementary Materials

The following supporting information can be downloaded at: https://www.mdpi.com/article/10.3390/nu14245220/s1, Figure S1: Association of total gestational weight gain with heart rate variability parameters of 2-year-old offspring in the whole cohort; Table S1: Heart rate variability parameters; Table S2: Gestational weight gain according to IOM recommendations; Table S3: Association of last trimester gestational weight gain on heart rate variability and heart rate in 2-year-old children of mothers with and without GDM.
