# Supplementary material for: Maternal Weight Gain during Pregnancy and the Developing Autonomic Nervous System—Possible Impact of GDM
Source: Nutrients. 2022 Dec 7;14(24):5220. doi: 10.3390/nu14245220 (PMC9784678; doi:10.3390/nu14245220)
Supplement: Supplementary file 1 [file nutrients-14-05220-s001.zip › nutrients-2019205-supplementary.pdf]

Table S1. Heart rate variability parameters.

**Variable (unit)**	**Description**	**Associated ANS function**
HR (bpm)	Heart rate, number of heart beats per minute	Sympathetic and parasympathetic
RMSSD (ms)	Root Mean Square of successive differences	Parasympathetic
SDNN (ms)	Standard Deviation of NN-Intervals (SDNN)	Overall HRV, sympathetic and parasympathetic
LF(m^2)	Absolute Power in low frequency band (0.04 - 0.15 Hz)	Sympathetic and parasympathetic
HF (m^2)	Absolute power in high frequency band (0.15 - 0.4 Hz)	Mainly parasympathetic
LF_n. u.	Relative power in the low frequency band in normalized units	Sympathetic and parasympathetic
HF_n. u.	Relative power in the high frequency band in normalized units	Mainly parasympathetic
LF/HF	LF-to-HF-ratio	Sympathovagal balance

Table S2. Gestational weight gain according to IOM recommendations (1).

Pre-pregnancy BMI (kg/m²)		Recommended weight gain (IOM) (kg)
Underweight	< 18.5	12.5 - 18
Normal weight	18.5 - 24.9	11.5 - 16
Overweight	25 - 29.9	7 - 11.5
Obese	> 30	5 - 9

1. Institute of Medicine (US) and National Research Council (US) Committee to Reexamine IOM Pregnancy Weight Guidelines. *Weight Gain During Pregnancy: Reexamining the Guidelines*. (Rasmussen KM, Yaktine AL, eds.). National Academies Press (US); 2009. Accessed April 8, 2019. http://www.ncbi.nlm.nih.gov/books/NBK32813/.

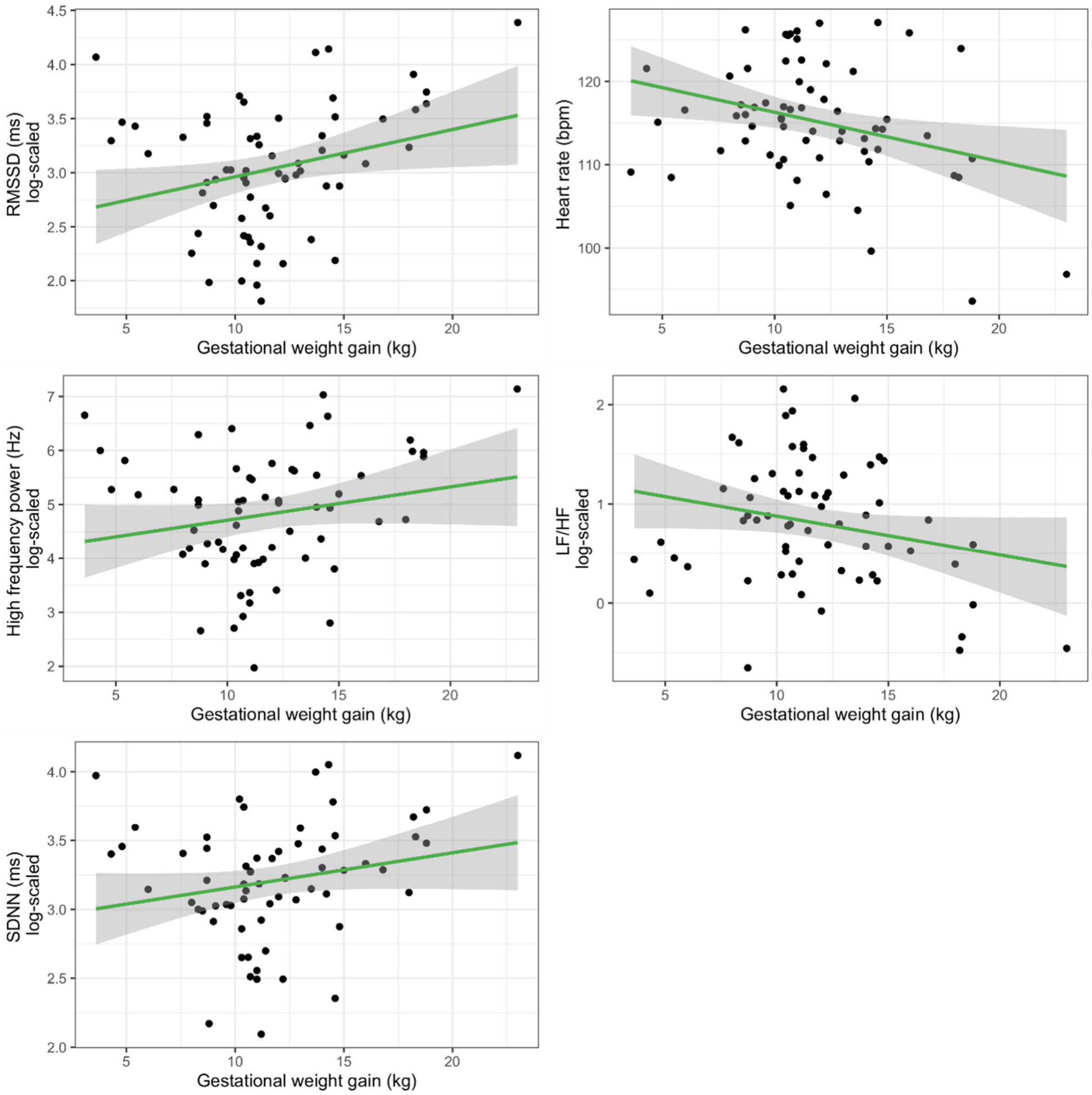

Figure S1. Association of total gestational weight gain with heart rate variability parameters of 2-year-old offspring in the whole cohort.

Table S3. Association of last trimester gestational weight gain on heart rate variability and heart rate in 2-year-old children of mothers with and without GDM .

	Heart rate (BPM)		RMSSD (ms)		SDNN (ms)		Low frequency (LF) power (m²)		High frequency (HF) power (m²)		LF_n. u.		HF_n. u.		LF/HF	
	**B (95% CI)**	**p**	**B (95% CI)**	**p**	**B (95% CI)**	**p**	**B (95% CI)**	**p**	**B (95% CI)**	**p**	**B (95% CI)**	**p**	**B (95% CI)**	**p**	**B (95% CI)**	**p**
**All children Interaction GWG x GDM-status**	1.91 (0.69, 3.14)	**0.0028**	-0.13 (-0.23, -0.03)	**0.0146**	-0.09 (-0.17, -0.01)	**0.0337**	-0.11 (-0.26, 0.03)	0.1116	-0.22 (-0.42, -0.01)	**0.0373**	2.01 (-0.29, 4.32)	*0.0856*	-1.99 (-4.27, 0.29)	*0.0852*	0.10 (-0.01, 0.21)	*0.0663*
**Children whose mothers had NGT**	-1.34 (-2.24, -0.44)	**0.0050**	0.10 (0.02, 0.17)	**0.0129**	0.05 (0.00, 0.10)	*0.0695*	0.02 (-0.07, 0.12)	0.6280	0.13 (-0.01, 0.27)	*0.0589*	-2.20 (-3.84, -0.56)	**0.0101**	2.16 (0.55, 3.77)	**0.0102**	-0.11 (-0.19, -0.03)	**0.0073**
**Children whose mothers had GDM**	0.69 (-0.20, 1.58)	0.1214	-0.05 (-0.12, 0.03)	0.1871	-0.05 (-0.11, 0.02)	0.1595	-0.10 (-0.22, 0.02)	0.1024	-0.11 (-0.28, 0.06)	0.1983	0.16 (-1.61, 1.93)	0.8544	-0.17 (-1.93, 1.59)	0.8423	0.01 (-0.08, 0.09)	0.8504

Effect sizes (95% CI) and p-values of multivariate linear regression models for the association of last trimester gestational weight gain (GWG) with offspring heart rate (HR) and heart rate variability (HRV). Row 3 shows the values for the interaction of total GWG and GDM-status with HR and HRV parameters, adjusted for pre-gestational BMI and offspring sex. Row 4 and 5 shows the values from multivariate linear regression of total GWG with HR and HRV parameters adjusted for pre-gestational BMI and offspring sex in groups of NGT and GDM separately. Bold values indicate statistical significance, italic values indicate statistical trend.
